# Oxygen Saturation Increase in Ischemic Wound Tissues after Direct and Indirect Revascularization

**DOI:** 10.3390/biomedicines12020367

**Published:** 2024-02-05

**Authors:** Austėja Račytė, Gabija Pikturnaitė, Tomas Baltrūnas, Evaldas Kalvaitis, Gediminas Vaitėnas, Arminas Skrebūnas, Vaida Baltrūnienė, Kęstutis Ručinskas

**Affiliations:** 1 Faculty of Medicine, Vilnius University, 01513 Vilnius, Lithuania; gabija.pikturnaite@mf.stud.vu.lt (G.P.); tomas.baltrunas@mf.vu.lt (T.B.); gediminas.vaitenas@mf.vu.lt (G.V.); arminas.skrebunas@mf.vu.lt (A.S.); vaida.baltruniene@mf.vu.lt (V.B.); kestutis.rucinskas@mf.vu.lt (K.R.); 2 Health Telematics Science Institute, Kaunas University of Technology, 44249 Kaunas, Lithuania; evaldas.kalvaitis@ktu.lt

**Keywords:** ischemic wounds, chronic limb-threatening ischemia, chronic total occlusion, angiosome, near-infrared spectroscopy, direct revascularization, indirect revascularization

## Abstract

Background: The primary approach for treating ischemic wounds is restoring oxygen supply to the ischemic region. While direct angiosomal revascularization is often associated with better post-operative wound healing and limb salvage, its superiority over non-angiosomal revascularization remains controversial. This study aimed to compare intraoperative tissue oxygen saturation changes in ischemic zones following either direct or indirect revascularization in below-the-knee arteries. Methods: This prospective observational study included patients undergoing direct and indirect below-the-knee endovascular revascularizations. Assignment to the groups was not randomized. Near-infrared spectroscopy was used to monitor rSO2 changes near the ischemic wounds intraoperatively. The changes were compared between the groups. Results: 15 patients (50%) underwent direct angiosomal revascularization, while an equal number of patients underwent indirect revascularization. Overall, a statistically significant increase in regional oxygen saturation was observed after revascularization (*p* = 0.001). No statistically significant difference was found between the direct and indirect revascularization groups (*p* = 0.619). Conclusions: This study revealed a minor difference in the oxygen saturation increase between the angiosomal and non-angiosomal revascularization groups. Such a finding indicates that the clinical significance of angiosomal revascularization is negligible and might be concealed by confounding factors, such as the vessel diameter and outflow impact on the restenosis rate.

## 1. Introduction

Despite many years of progress and scientific innovations in the field of wound healing, the burden of chronic wounds still has a relevant impact on healthcare costs and resource consumption [1,2]. It is reported that in the USA alone, chronic lower limb wounds affect up to 4.5 million people [3], while in developed countries, the financial commitment to care for such wounds makes up to 3% of the overall healthcare budget [4]. Notably, chronic wounds derive from various causative factors, with vascular pathologies, particularly arterial and venous leg ulcers, standing out as the most prevalent and economically impactful [5]. Chronic wounds significantly contribute to reduced life quality and cause impairment or loss of function and even death [3]. Among the spectrum of chronic wounds, ischemic wounds induced by peripheral arterial disease (PAD) are characterized by the highest reported mortality rate [6]. However, regardless of the specific etiology of the wound, one of the most crucial elements for wound healing is the state of the vasculature. Inadequate tissue perfusion disrupts the supply of nutrients and other factors that are critically important for wound healing, leading to reduced collagen deposition, impaired angiogenesis, and poor epithelialization [7]. Under physiological conditions, relative hypoxia induces angiogenesis and activates several important factors, such as vascular endothelial growth factor (VEGF), transforming growth factor-beta (TGF-β), and platelet-derived growth factor (PDGF), which are vital elements for the wound-healing process [8]. However, in cases of severe ischemia, the response to hypoxia becomes aberrant, causing dysfunctions in protein induction pathways and ultimately resulting in the formation of non-healing wounds [5]. Therefore, the primary approach to healing arterial wounds is restoring the blood supply to the ischemic region, which can be achieved by performing open surgical repair and endovascular interventions [9]. The latter method gives an opportunity to revascularize not only one but several different arteries during the same intervention. Moreover, the endovascular technique allows for restoring the blood flow directly to the artery supplying the ischemic zone, based on the angiosome concept, even when the run-off vessel is very poor [10]. However, the clinical effect of revascularizing a vessel with a poor run-off still remains undetermined.

Direct angiosomal revascularization is reported to have better post-operative wound healing and limb salvage results [11,12]. Therefore, in clinical practice, it is recommended to consider performing direct revascularization first in patients with significant wounds [9]. It is also suggested that direct revascularization is especially valuable for patients with diabetic foot ulcers since they are known to have poorly developed infrapopliteal arterial collaterals [13], while in the presence of good collateral vessels, direct revascularization loses its primary value [14]. Moreover, performing direct revascularization is not always achievable, due to incompatible anatomy or technical reasons. When angiosomal revascularization is not possible, the only option to improve blood perfusion in ischemic tissues is to revascularize the most technically approachable artery with the best run-off. The decision to perform indirect revascularization as well as choosing the most suitable artery for revascularization in different clinical scenarios fully depend on the doctor’s opinion. Despite endovascular below-the-knee revascularizations being widely performed in everyday clinical practice, there is still debate about whether direct angiosomal revascularization is superior to indirect [15].

While it has been established that revascularization is the most straightforward pathway for healing arterial chronic wounds, the lack of a suitable method to quantitatively assess the efficacy of revascularization, to this day, remains a challenge. Traditionally, the success of revascularization has been evaluated by endpoints such as wound healing and limb salvage rates [16]. However, these are very distant indicators of procedural success. Therefore, intraoperative methods for assessing the success of revascularization are needed. Currently, the evaluation of revascularization success often relies on subjective assessments, such as the observation of present wound blush in post-revascularization angiography, which is believed to correlate with an increased likelihood of better wound healing [17]. Yet, the subjectivity of such assessment limits its reliability. A new potential approach for the assessment of the procedural success could be intraoperative tissue perfusion measurement. The existing methods for perfusion measurement include hyperspectral imaging [18], 2D perfusion angiography [19], micro-oxygen sensors (MOXYs) [20], skin perfusion pressure measurement [9], transcutaneous oxymetry (TcPO2), and near-infrared spectroscopy (NIRS) [21]. However, most of these methods exhibit inconsistency and are influenced by various factors, such as temperature and vasospasm, making them unsuitable for intraoperative applications. For instance, transcutaneous oximetry, although featuring lower variability due to its proprietary heating system, poses limitations in intraoperative monitoring due to possible skin burns. In addition, transcutaneous oxymetry interferes with X-ray imaging due to the significant amount of metal alloys in the detector. Given these considerations, near-infrared spectroscopy (NIRS) emerges as a preferable choice for intraoperative tissue perfusion monitoring in this study. This selection is based on the relatively straightforward applicability of NIRS, aiming to address the limitations posed by other existing perfusion measurement methods.

The aim of this study was to evaluate and compare intraoperative oxygen saturation changes in ischemic wound regions after performing either indirect or direct angiosomal revascularization in below-the-knee arteries.

## 2. Materials and Methods

### 2.1. Study Type and Ethics

We performed a non-randomized prospective observational study at a single center, the Vilnius City Clinical Hospital. This clinical trial was reviewed and approved by the Vilnius Regional Biomedical Research Ethics Committee on the 5 December 2017, registration number 158200-17-981-482. On 2 April 2019, the study was registered in clinicaltrials.gov, registration number NCT03898869. Each participant signed an informed consent form before any study-related procedure.

### 2.2. Participants

All participants had to meet the inclusion and exclusion criteria depicted in Table 1. Only CLTI (Rutherford V–VI) patients with chronic total occlusion in below-the-knee arteries that were scheduled for treatment were included in this clinical trial.

### 2.3. Examination and Procedures

All patients underwent routine laboratory and clinical assessments. Ischemic wounds were evaluated using WIfI classification [22]. The Dopplex^®^ Ankle Brachial Pressure Index Kit with an EZ8 8 MHz Probe, Huntleigh (Cardiff, Wales, UK) was used for the ankle–brachial index measurement. Either direct or indirect endovascular revascularization was carried out in all cases. The only intended revascularization technique was percutaneous transluminal angioplasty (PTA), and stenting was a bailout option in flow-limiting dissections. The operating room was equipped with the Innova 4100, GE (Boston, MA, USA). The oxygen saturation in the index finger and vital signs was registered using the B40 Patient Monitor, GE (Boston, MA, USA). Endovascular procedures were performed by a single vascular surgeon according to the local procedure protocol, with heparinization during the procedure and a prescription of dual antiplatelet therapy for 3 months after the intervention. Patient assignment to direct or indirect revascularization groups was not randomized and was performed by the operating doctor based on the vessel size, occlusion length, and outflow. If several arteries, including the angiosomal vessel, were successfully revascularized, the patients were allocated to the direct revascularization group.

**Figure 1:**
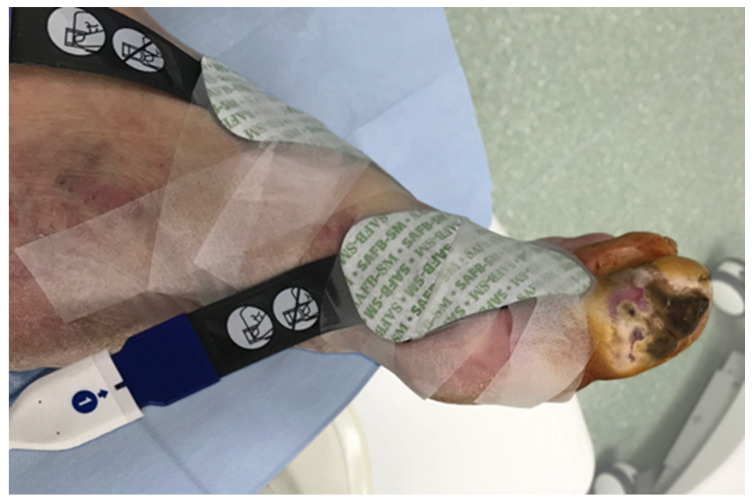
NIRS sensor placement near the ischemic wound.

Tissue oxygen saturation changes were measured intraoperatively with near-infrared spectroscopy (NIRS) using the Invos Oximeter, Somanetics/Medtronic (Dublin, Ireland). Two sensors were located on the healthy skin 2–3 cm from the ischemic wound, and one reference probe was placed on the pectoral muscle (Figure 1). During the endovascular procedure, regional oxygen saturation (rSO2) changes could be seen on the screen of the Invos Oximeter and were recorded every 6 s.

After every procedure, the NIRS data was downloaded and post-processed using Excel v16.42, Microsoft (Redmond, WA, USA). Afterwards, the means of the first and last 50 measurements of every sensor were calculated. The formula below was used to measure the revascularization effect.

$$Effect=\left(\frac{\frac{\left(M_{1\alpha }+M_{2\alpha }\right)-\left(M_{1\omega }+M_{2\omega }\right)}{2}-\left(MR_{\alpha }-MR_{\omega }\right)}{\frac{M_{1\alpha }+M_{2\alpha }}{2}}-1\right)*100$$

where:

M_1α_—mean of the first 50 measurements on sensor 1 (before revascularization);
M_2α_—mean of the first 50 measurements on sensor 2 (before revascularization);
M_1ω_—mean of the last 50 measurements on sensor 1 (after revascularization);
M_2ω_—mean of the last 50 measurements on sensor 2 (after revascularization);
M_Rα_—mean of the first 50 measurements on the reference sensor (before revascularization);
M_Rω_—mean of the last 50 measurements on the reference sensor (after revascularization).

### 2.4. Statistical Analysis

Statistical analysis was carried out using SPSS v26.0, IBM (Armonk, NY, USA). The data are presented as the mean ± SD for the continuous values, which were distributed normally, otherwise the median and interquartile range (25th and 75th percentiles) are shown (IQR). Student’s *t*-test was used to assess the statistical significance of normally distributed data, the Wilcoxon signed rank test was used for continuous non-normally distributed variables, and Fisher’s exact test was used for categorical variables. Differences among the samples were considered statistically significant when *p* ≤ 0.05.

## 3. Results

This clinical trial included 30 patients with chronic limb-threatening ischemia (Rutherford V–VI) and chronic total occlusion in below-the-knee arteries. A total of 17 (57%) out of the 30 patients were male. The mean age of the patients was 74.7 ± 11.2 years. The baseline characteristics of both the direct and indirect revascularization groups are depicted in Table 2. No statistically significant difference between the groups was found regarding the baseline characteristics.

Lesions in all three angiosomes of the foot were observed in this clinical trial. The wound localization is presented in Table 3. A total of 16 patients had lesions in the anterior tibial artery angiosome, 8 patients had them in the posterior tibial artery, and 6 patients had them in the peroneal artery angiosome. 

Every patient had angiographically verified lesions in all three below-the-knee arteries. In total, 30 endovascular below-the-knee procedures were performed, and 44 arteries were revascularized. The revascularization locations were as follows: 21 anterior tibial arteries, 10 posterior tibial arteries, 8 peroneal arteries, 2 tibioperoneal trunks, and 3 popliteal arteries. A total of 15 patients (50%) underwent direct angiosomal revascularization, and the other half of the patients underwent indirect revascularization.

The NIRS revealed a statistically significant intraoperative rSO2 increase near the wound after revascularization (paired samples *t*-test, *p* = 0.001) (Table 4).

**Figure 2:**
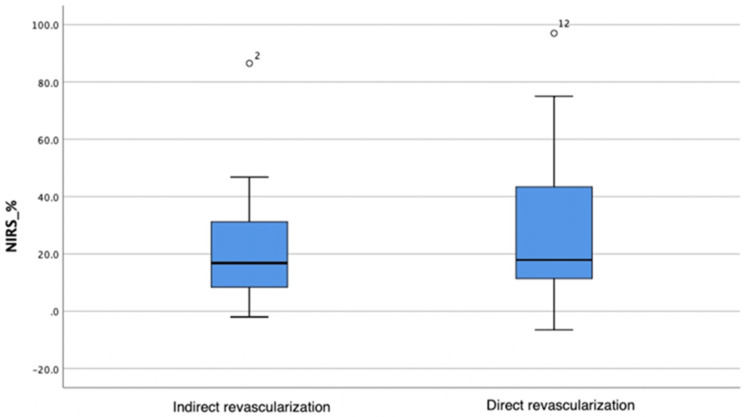
Oxygenation changes in the indirect and direct angiosomal revascularization groups. The horizontal bar represents the median value, while the blue box spans the interquartile range (IQR), representing the 25th to 75th percentiles. Whiskers represent minimum and maximum values. Outliers are represented separately as number 2 and number 12.

A greater oxygen saturation increase was observed in the direct angiosomal revascularization group; however, the difference in change between the groups was not statistically significant (Mann–Whitney test, *p* = 0.619) (Table 5; Figure 2).

### Post-Hoc Power Analysis

A post-hoc power analysis showed that, for an independent group *t*-test with a significance level of 0.05 and a power of 0.95, the required sample size was 884 subjects.

## 4. Discussion

Despite the fact that wound management was discussed for the first time more than 4000 years ago, the burden of chronic wounds still remains very high and accounts for around 40 million patients worldwide [23]. The prevalence of chronic wounds is notably higher among the elderly population, and with the current global demographic shift toward an increasing proportion of elderly individuals, it is anticipated that the incidence of chronic wounds will correspondingly escalate. It is also important to emphasize that especially in the elderly, chronic wounds are prone to be rather multi-etiological [2]. This complicates the assessment of the arterial component, necessitating a specific approach that incorporates the measurement of tissue perfusion in the proximity of the wound site. Since the existing methods are suboptimal, we believe that future prospects for wound evaluation and management will likely include novel methods for tissue perfusion monitoring. This study shows that, despite the limited feasibility of NIRS, under highly controlled conditions, perfusion changes in tissues near ischemic wounds could be monitored intraoperatively during both direct angiosomal and non-angiosomal revascularization procedures. These findings open new prospects for the further exploration of NIRS as a viable tool in the setting of chronic wound evaluation. 

To this day, intraoperative quantitative evaluation of reperfusion remains controversial. The only validated tool for measuring perfusion in tissue is transcutaneous oximetry (TcPO2) [24]. However, the application of TcPO2 requires skin heating to 40 °C, is operator-dependent, time-consuming, and impacts X-ray imaging, which makes it not suitable for intraoperative tissue perfusion monitoring [9].

In this clinical trial, near-infrared spectroscopy (NIRS) was used to monitor tissue oxygen saturation changes in ischemic wound regions during angiosomal and non-angiosomal revascularization procedures in below-the-knee arteries. The NIRS system comprises a monitor and flexible optodes equipped with a light source and two receiving photodetectors. The basic functioning involves the generation of near-infrared (NIR) light at specific wavelengths, which are absorbed by tissue hemoglobin. This emitted NIR beam is directed into the target tissue through cutaneously attached optodes. Subsequently, the NIRS system determines the proportion of oxygenated hemoglobin within small vessels by analyzing the amount of detected light in the photodetectors. Notably, the NIRS method, with its spectral range spanning from 700 to 1100 nm, exhibits the ability to penetrate tissue to depths several centimeters beyond the reach of visible light [25,26]. It is an easy-to-apply, non-invasive perfusion measurement method that does not interfere with X-ray imaging significantly, is not harmful to the tissue, and therefore, can be used intraoperatively for extended period of time [27]. The INVOS^TM^ regional oximeter has been validated to monitor brain perfusion during coronary artery bypass surgeries. However, the importance of this method is growing in different clinical applications [28]. Over the past 10 years, there have been 67 papers published in PubMed regarding the use of NIRS for brain perfusion monitoring during carotid endarterectomies (CEA), which implies that, due to its simplicity, NIRS is being widely adopted in this clinical setting [29,30]. Recently published clinical trials have reported the utilization of NIRS for peripheral tissue oxygen saturation monitoring in PAD patients [31,32,33]. Also, in 2022, Baltrunas et al. discussed the use of NIRS in the context of PAD. In their systematic review, the authors reported NIRS as a promising tissue perfusion measurement tool, particularly in diabetic patients; however, it was stated that more structured clinical data are needed in order to evaluate the effectiveness of this method in peripheral tissue oxygen saturation measurement for PAD patients [27]. Therefore, taking into account the previously carried out clinical trials as well as the existing literature, we believe that NIRS conforms well to the design of our study. 

The angiosome concept was first introduced in 1987 by Ian Taylor and colleagues. There are six angiosomes in the foot, which originate from three main arteries: the anterior tibial artery, posterior tibial artery, and peroneal artery [10,33]. The angiosome concept is primarily based on the anatomy of healthy limbs and has been adopted in the field of plastic surgery. However, currently, it is being used in CLTI limbs with long-term CTO and remodeled vascular anatomy [11,12]. In their systematic review and meta-analysis, Dilaver et al. found that direct revascularization leads to better wound healing and limb salvage results [11]. On the other hand, indirect revascularization might appear beneficial when significant collateral vessels are present. Varela et al. found that post-operative outcomes after the restoration of blood flow to the ischemic area through collaterals are similar to those after direct revascularization [34]. However, in patients with diabetes and renal function impairment, collaterals are usually not well formed, making the direct technique more appropriate [15]. Despite the growing evidence supporting revascularization according to the angiosome concept, the literature comparing indirect versus direct blood flow restoration is considered to be low-quality. Moreover, it measures outcomes such as wound healing and limb salvage results, which are surrogate indicators of blood perfusion restoration, early artery recoil or later restenosis, inflammation, wound depth, and other comorbidities [11,15]. 

Existing studies, as well as our clinical trial, compare revascularization results between two major groups, which are based only on the angiosomal and non-angiosomal approaches (groups 1 and 2) (Table 6) [13,35,36,37].

In clinical practice, there is no question that directly revascularizing the anatomically optimal vessel (subgroup 1a) (Table 6) will result in the best post-operative outcome. Also, indirect revascularization of a suboptimal vessel with impaired run-off and a small diameter (subgroup 2b) (Table 6) will lead to the poorest result. However, to this day, there is a lack of discussion to verify which clinical scenario leads to better results: the direct angiosomal revascularization of a poor-outflow vessel (subgroup 1b) or the indirect revascularization of an optimal artery (subgroup 2a). Therefore, it can be presumed that there is a need for studies comparing the revascularization results between only the subgroups 1b and 2a, which remain controversial in clinical practice. 

Even though our clinical trial is the largest trial to date investigating intraoperative angiosomal versus non-angiosomal revascularization results using NIRS, our sample was too small to divide the patients into the four previously mentioned subgroups. Future clinical trials evaluating and comparing revascularization outcomes among these less straightforward patient subgroups could potentially help gather higher-quality data on the use of the angiosome concept and would shed some light on the ongoing debates on whether direct angiosomal revascularization is superior to indirect. Also, a larger multi-center study including wound healing and limb salvage results could be beneficial.

Our study reveals only a minor difference in the rSO2 increase between the angiosomal and non-angiosomal revascularization groups (17.9% and 16.8% increases in tissue oxygen saturation, respectively). Moreover, the post-hoc power analysis showed that a very large sample of approximately 900 patients is needed to obtain a statistically significant difference between the aforementioned groups. This indicates that the difference between angiosomal and non-angiosomal revascularization is extremely small and shadowed by other variables, such as early recoil, later restenosis of treated arteries, wound depth, inflammation, etc. In addition, the existing large randomized clinical trials concerning revascularization success take into account many other factors, such as patient comorbidities or the type of debulking devices/balloons/stents used, which appear to be more influential in this clinical setting. 

Being the first of this kind, this study has some limitations, such as the absence of patient randomization, which could have caused selection bias. Patient assignment to direct or indirect revascularization group was performed solely by the operating doctor based on angiographic image evaluation and the doctor’s experience in this field. However, in every case, the revascularization method selection was adequate for the patient. Therefore, our results might have been affected slightly more by the operator’s level of clinical expertise rather than the differences in revascularization technique. 

Randomization for this type of study would need a significantly higher number of participants. However, not all occluded BTK vessels can be opened equally successfully, and there would be a huge shift among the groups for the intended treatment and actual revascularization. In this case, the sample size was too small to efficiently stratify patients regarding their angiographic baseline characteristics, MAC-SAD score, and other existing scoring systems. In this study, WIfI classification was used to assess the ischemic wounds of all participants. However, this classification itself has more possible combinations than the sample size of this study. Hence, we decided to not stratify the patients according to this classification as well.

## 5. Conclusions

Although this study confirmed a significant tissue oxygen saturation increase using NIRS near ischemic wounds after revascularization, only a minor difference in the oxygen saturation increase between the direct and indirect revascularization groups (17.9% and 16.8% increases in the tissue oxygen saturation, respectively) was observed. Consequently, this study indicates that the clinical significance of angiosomal revascularization is negligible and most likely concealed by the vessel diameter and outflow impact on the restenosis rate. We believe that future studies comparing the outcomes only between suboptimal angiosomal and optimal non-angiosomal revascularization subgroups are needed. Such clinical trials would guide doctors through clinical situations that, to this day, remain controversial. Furthermore, adequate intraoperative perfusion measurement methods would provide a chance to predict the success of revascularization while still being in the operating room. This would respectively lead to better patient outcomes, including more efficient wound healing, consequently contributing to a global reduction in the economic burden imposed by chronic wounds.

## Figures and Tables

**Table 1:** Inclusion and exclusion criteria.

Inclusion Criteria	Exclusion Criteria
All presenting PAD patients 55–95 years old	Skin diseases preventing the use of NIRS
CLTI Rutherford V–VI	Life expectancy less than 12 months
CTO below the knee	Unavoidable amputation above ankle
Planned revascularization of at least one BTK artery	Blood oxygen saturation below 85% because of any comorbidities
No need for intervention above the knee	

CLTI—critical limb-threatening ischemia; CTO—chronic total occlusion; NIRS—near-infrared spectroscopy; PAD—peripheral artery disease; BTK—below the knee.

**Table 2:** Baseline characteristics of direct and indirect revascularization groups.

Variables	Direct Revascularization Group	Indirect Revascularization Group	*p*-Value
Age, years	72.3 ± 7.8	77.1 ± 13.7	>0.05
Male	7 (46.7%)	10 (66.7%)	>0.05
Diabetes mellitus	10 (66.7%)	6 (40%)	>0.05
End-stage renal disease	6 (40%)	4 (26.7%)	>0.05
CAD	12 (80%)	9 (60%)	>0.05

CAD—coronary artery disease. Data are presented as *n* (%) and as mean ± standard deviation.

**Table 3:** Ischemic wound localization according to angiosome.

	Lesions (*n* = 30)
Anterior tibial artery	16 (53.3)
Posterior tibial artery	8 (26.7)
Peroneal artery	6 (20.0)

Data are presented as *n* (%).

**Table 4:** Intraoperative rSO2 increase in sensors 1 and 2 located near the wound.

Sensor	NIRS rSO2 before the Reperfusion	NIRS rSO2 after the Reperfusion	*p*-Value
**Sensor 1**	58.0 ± 12.7	66.7 ± 11.6	0.001
**Sensor 2**	57.6 ± 12.7	67.1 ± 14.0	<0.001

Data are presented as mean ± standard deviation.

**Table 5:** Oxygenation changes in the indirect and direct angiosomal revascularization groups.

	Patients (*n* = 30)	NIRS rSO2 Change after the Revascularization
**Indirect** **Revascularization**	15 (50)	16.8 [25.7]
**Direct** **Revascularization**	15 (50)	17.9 [38.5]

Data are presented as *n* (%) and median [interquartile range, IQR].

**Table 6:** Proposed formation of subgroups.

	Optimal Vessel (a)	Suboptimal Vessel (b)
**Angiosomal revascularization (1)**	Subgroup 1a	Subgroup 1b
**Non-angiosomal revascularization (2)**	Subgroup 2a	Subgroup 2b

## Data Availability

Dataset available on request from the authors.
